# Infliximab (IFX)-Biosimilar Induced Drug-Induced Liver Injury (DILI): A Case Report

**DOI:** 10.7759/cureus.32525

**Published:** 2022-12-14

**Authors:** Maria Zachou, Konstantinos Pikramenos, Maria Panoutsakou, Efthimia Lalla, Theodoros Androutsakos

**Affiliations:** 1 Second Department of Propaedeutic Surgery, "Laikon" General Hospital, Medical School, National and Kapodistrian University of Athens, Athens, GRC; 2 Gastroenterology, Sismanogleio General Hospital of Athens, Athens, GRC; 3 Second Department of Urology, National and Kapodistrian University of Athens, Sismanoglio General Hospital, Athens, GRC; 4 Gastroenterology, Sismanogleio General Hospital, Athens, GRC; 5 Internal Medicine, National and Kapodistrian University of Athens School of Medicine, Athens, GRC

**Keywords:** crohn's disease, infliximab ct-p13, hepatotoxicity, dili, infliximab biosimilar

## Abstract

Infliximab (IFX) is a chimeric human-murine monoclonal antibody that prevents tumor necrosis factor alpha (TNF-α) activation by binding to both soluble and transmembrane forms of TNF-α. Antagonists of TNF (anti-TNF agents) can cause drug-induced liver injury (DILI). We present a non-anti-TNF naïve patient suffering from severe Crohn’s disease who developed DILI with a hepatocellular pattern, without jaundice, after two infusions of an IFX biosimilar.

## Introduction

Infliximab (IFX) is a monoclonal antibody that neutralizes the human tumor necrosis factor alpha (TNF-α). It is used for induction and maintenance of disease remission, in patients with known Crohn’s disease or ulcerative colitis [1]. Commonly reported side effects include serious bacterial and fungal infections, reactivation of hepatitis B virus, vasculitis and various malignancies, especially hepatosplenic T-cell lymphoma [2-3]. Idiosyncratic DILI is a somewhat rare side effect and remains underreported despite its seriousness. The most commonly reported IFX-induced DILI is the transient elevation of transaminases that only resolves after drug discontinuation. Cases of drug-induced autoimmune hepatitis and secondary sclerosing cholangitis have also been reported [4-5].

Infliximab was first authorized in the European Union (EU) in 1998 under the invented name of Remicade (originator infliximab, IFX-O) [6]. After patents expired, biosimilars were introduced. According to the World Health Organization definition, biosimilars are biotechnological products that are shown to be highly similar in terms of their quality, safety, and efficacy to an already licensed reference product [7]. The first IFX biosimilar was CT-P13 and was approved in the EU in September 2013 [8].

The authors aim to present the second case of DILI after infusion of IFX biosimilar CT-P13. As biosimilars are widely used, it is imperative that clinicians are aware of this potential side effect.

## Case presentation

We present a 42-year-old woman with a known medical history of nephrolithiasis and severe isolated colonic Crohn’s disease (Montreal A2L2B1), first diagnosed in 2018. She had previously received vedolizumab (integrin antagonist) and ustekinumab (interleukin 12/23 antagonist) without any clinical response and ultimately underwent total colectomy with ileorectal anastomosis in 2020. She was then treated with adjuvant treatment with adalimumab (an anti-TNF agent) and subsequently developed ileal Crohn’s disease, with multiple disease relapses in a two-year period and five total treatments of IV corticosteroids. In 2022, due to poor response to adalimumab, a change of treatment was decided and a biosimilar of IFX, CT-P13 was initiated (an anti-TNF agent). After comprehensive laboratory testing before each infusion, she received the first IV dose of 5 mg/kg (275 mg) on May 11th and a second dose on May 26th.

Ten days after the second infusion, she presented in the emergency department, complaining of extreme fatigue, anorexia, nausea, and vomiting that started 24 h before. Clinical examination revealed mild diffuse abdominal pain, without any other clinical signs. She reported no alcohol intake or over-the-counter drugs in the last months and her laboratory testing indicated acute liver injury with a hepatocellular pattern (Table 1). The patient underwent abdominal ultrasound with Doppler and CT in order to exclude focal changes in the liver parenchyma, biliary disease, portal vein thrombosis, and Budd-Chiari syndrome; both revealed no abnormalities. Due to a lack of biochemical evidence of an impending liver failure, the patient was discharged and did not receive any corticosteroid treatment, according to EASL guidelines [9]. As DILI is a diagnosis of exclusion, further diagnostic work-up for viral and autoimmune hepatitis, Wilson disease, hemochromatosis, and alpha-1-antithrypsin deficiency were performed; all of them were negative (Table 2). A liver biopsy was recommended but the patient denied it. The RUCAM (Roussel Uclaf Causality Assessment Method) score was 15.50. A clinical diagnosis of IFX-induced DILI was made so IFX was stopped and a watchful waiting approach was decided. The patient’s aspartate aminotransferase (AST) and alanine transaminase (ALT) serum levels gradually improved and normalized two months later.

**Table 1:** Laboratory tests performed at the emergency department. WBC, white blood cells; Hb, hemoglobin; PLTs, platelets; INR, international normalized ratio; APTT, activated partial thromboplastin time; ALT, alkaline aminotransferase; AST, aspartate aminotransferase; GT, glutamyl transferase; LDH, lactate dehydrogenase; ALP, alkaline phosphatase; CPK, creatine phosphokinase; CRP, C-reactive protein

Lab test	Result	Reference value	Unit
WBC	13.00	4.00–11.00	K/μL
Neutrophils	45.2	40.0–75.0	%
Lymphocytes	40.5	20.0–40.0	%
Hb	15.3	13.5–17.5	g/dL
PLTs	193	150–400	K/μl
INR	0.98	0.90–1.15	-
APTT	24.31	21.0–33.90	s
Glucose	97	60–100	mg/dL
Urea	30	10–50	mg/dL
Creatinine	1.1	0.5–1.5	mg/dL
Albumin	4.8	3.5–5.1	g/dL
ALT	234	4–45	U/L
AST	265	4–45	U/L
γGT	17	5–45	U/L
LDH	145	135–225	U/L
ALP	43	42–128	U/L
CPK	129	25–190	U/L
Total bilirubin	0.53	0.20–1.40	mg/dL
Direct bilirubin	0.23	0.00–0.50	mg/dL
Indirect bilirubin	0.30	0.10–0.90	mg/dL
CRP	0.9	<6	mg/l
Sodium	143	135–148	mEq/L
Potassium	4.2	8–40	mEq/L

**Table 2:** Further work-up. Anti-HAV IgM, antibody to hepatitis A virus (immunoglobulin M); HBsAg, hepatitis B surface antigen; Anti-HBs, antibody to the hepatitis B surface antigen; Anti-HBc, antibody to the hepatitis B core antigen; Anti-HCV, antibody to hepatitis C virus; Anti-HDV, antibody to hepatitis D virus; Anti-HEV IgM, antibody to hepatitis E virus (immunoglobulin M); HIV, human immunodeficiency virus; Anti-HSV IgM, antibody to herpes simplex virus (immunoglobulin M); anti-CMV IgM, antibody to cytomegalovirus (immunoglobulin M); Anti-EBV IgM, antibody to Epstein-Barr virus (immunoglobulin M); Anti-VZV IgM, antibody to varicella-zoster virus (immunoglobulin M); ANA, antinuclear antibody; AMA, anti-mitochondrial antibody; ASMA, anti-smooth-muscle antibody; LKM 1,3, liver kidney microsomial antibodies 1,3; p-ANCA, perinuclear anti-neutrophil cytoplasmic antibodies

Lab test	Result
Anti-HAV IgM	Negative
HBsAg	Negative
Anti-HBs	Positive
Anti-HBc	Negative
Anti-HCV	Negative
Anti-HDV	Negative
Anti-HEV IgM	Negative
HIV	Negative
Anti-HSV IgM	Negative
Anti-CMV IgM	Negative
Anti-EBV IgM	Negative
Anti-VZV IgM	Negative
ANA	Negative
AMA	Negative
ASMA	Negative
LKM-1	Negative
LKM-3	Negative
p-ANCA	Negative
A1 antitrypsin	Normal
Ceruloplasmin	Normal
Transferrin saturation	Normal

Due to limited available treatment options and a lack of definite diagnosis, the treating physician decided to follow a positive re-challenge strategy and prescribed IFX once again. She had received three infusions in total and on the seventh day after the last one, the patient’s transaminases started to rise again (ALT, 156 U/L; AST, 112 U/L). Thus, a diagnosis of IFX-induced DILI was made and treatment with IFX was terminated. Therapy with natalizumab (alpha 4 integrin inhibitor) was initiated and three months later, liver enzymes are within normal levels and no other adverse events have been reported. Six months later, her clinical response was adequate with no disease relapse.

## Discussion

Drug-induced liver injury is typically classified as idiosyncratic DILI, caused by agents that have little or no intrinsic toxicity and lead rarely to liver injury, and direct DILI caused by agents that are intrinsically toxic to the liver [10]. In idiosyncratic DILI the injury is unpredictable, not dose-dependent, and not reproducible in animal models, while in direct DILI the injury is common, predictable, dose-dependent, and reproducible in animal models [11]. DILI represents a very common cause of transaminasemia in developed countries, as well as the most common cause of acute liver injury in these countries, with acetaminophen poisoning being responsible for about 50% of all acute liver injury cases [12]. Almost every drug, used in practice, can lead to DILI, with anti-TNF agents being no exception to that. As a matter of fact, transaminasemia is one of the most common adverse effects of anti-TNF agents, especially of IFX, found in almost 6% of all patients with inflammatory bowel disease starting treatment with these drugs [13].

 Infliximab is the most common cause of DILI among all anti-TNF agents, and it seems to be hepatotoxic both via an autoimmune as well as via a direct mechanism [14]. Autoimmune-induced DILI seems to be the most common cause of severe IFX-related liver injury, accounting for more than 90% of all cases, characterized by the presence of multiple autoantibodies and autoimmune hepatitis in liver histolology. On the other hand, direct hepatotoxicity usually occurs after two to five infusions and is commonly transient and asymptomatic. Recovery typically occurs 4-12 weeks after treatment cessation. Severe transaminasemia is highly uncommon, occurring mainly in individuals with concomitant hepatotoxic drugs and/or underlying liver diseases [15].

CT-P13 is a biosimilar version (Janssen Biotech, Horsham, PA, USA) of the original anti-TNF medical agent IFX and it was approved by the European Medicines Agency (EMA) in September 2013 and by the US Food and Drug Administration (FDA) in April 2016 [16-17]. Biosimilars are known to have “no clinically meaningful differences in terms of safety, purity, and potency” with the original version [18]. In terms of treatment effects, there are several prospective and retrospective studies that have shown the non-inferiority of CT-P13 to IFX in patients with active Crohn’s disease. Regarding the safety profile and immunogenicity of the biosimilar, the results of these studies were encouraging as no differences have been revealed between the originator and biosimilar [19-20].

 According to our knowledge, this is the second case report of DILI after infusion of IFX biosimilar. The first case was publicated a few months ago, describing a 23-year-old woman with Crohn’s disease, who developed DILI after switching from original IFX to IFX biosimilar CT-P13. She underwent a liver biopsy that showed prominent pericentral canalicular cholestasis, without other findings related to steatosis or sclerosing cholangitis. These results were consistent with drug-induced cholestasis. The biosimilar CT-P13 was stopped and the patient improved 10 weeks after that. Crohn’s disease management continued with re-switching from CT-P13 to IFX-O without any liver-related adverse events [21].

Our patient has only received the biosimilar form of IFX (CT-P13). Furthermore, the patient had already received two other anti-TNF agents without reporting transaminasemia or other liver damage. Unfortunately, our patient did not undergo liver biopsy yet she had another episode of transaminasemia, shortly after her next IFX infusion, so we believe there is no doubt regarding her diagnosis.

## Conclusions

Despite it is not biopsy confirmed, we strongly believe that this case is the second case of IFX biosimilar-induced DILI in literature, probably caused due to idiosyncratic hepatotoxicity. Since CT-P13 biosimilar of IFX is a commonly used agent, we feel that physicians should be alert about patients under IFX treatment that develop.
